# CannabisGDB: a comprehensive genomic database for *Cannabis Sativa* L

**DOI:** 10.1111/pbi.13548

**Published:** 2021-02-04

**Authors:** Sen Cai, Zhiyuan Zhang, Suyun Huang, Xu Bai, Ziying Huang, Yiping Jason Zhang, Likun Huang, Weiqi Tang, George Haughn, Shijun You, Yuanyuan Liu

**Affiliations:** ^1^ Basic Forestry and Proteomics Center Haixia Institute of Science and Technology State Key Laboratory of Ecological Pest Control for Fujian and Taiwan Crops College of Forestry Fujian Agriculture and Forestry University Fuzhou China; ^2^ School of Life Sciences Capital Normal University Beijing China; ^3^ Xiamen ZeeMan Biotechnology Co., Ltd Xiamen China; ^4^ Key Laboratory of Genetics, Breeding and Multiple Utilization of Crops (FAFU) Ministry of Education Fujian Agriculture and Forestry University Fuzhou Fujian China; ^5^ Marine and Agricultural Biotechnology Laboratory Institute of Oceanography Minjiang University Fuzhou China; ^6^ Department of Botany University of British Columbia Vancouver British Columbia Canada; ^7^ Institute of Applied Ecology Fujian Agriculture and Forestry University Fuzhou China; ^8^ Joint International Research Laboratory of Ecological Pest Control Ministry of Education Fuzhou China

**Keywords:** database, functional genomic, transcriptomics, proteomics, metabolomics, cannabis

Being one of the world’s oldest crops with significant economic and medicinal importance, *Cannabis Sativa* L. (cannabis) has attracted an enormous amount of attention and become one of the most popularly cultivated plants worldwide (Gao *et al*., 2020). The content of Δ9‐tetrahydrocannabinol (THC) determines the legal status of the cannabis varieties. Uncontrolled varieties called hemp are defined as those that have 0.3% or less THC, while marijuana is defined as varieties with higher than 3% (4%–35%). Currently, hemp has been introduced to more than 130 countries with 94 certified hemp varieties for large‐scale cultivation as food, textiles and building materials based on the statistics from Organization for Economic Co‐operation and Development (OECD). On the other hand, medical marijuana has received considerable interest, thanks to their therapeutic effects and phytomedicinal use (Di Marzo, 2006).

The last decade has seen advancements in understanding cannabis genetics. In 2011, van Bakel et al. first published draft genome and transcriptome of Purple Kush (CsPK), a medicinal marijuana strain with an average THC level at 20% and a non‐detect level of cannabidiol (CBD) (van Bakel *et al*., 2011). The same group also reported the draft genome of a hemp variety Finola (CsFN) with an average CBD level at 7% and low THC (<0.3%). These reference genomes were recently updated by combining third‐generation sequencing and genetic map data, resulting in 20 chromosomes with hundreds of distinct repeat sequence families (Laverty *et al*., 2018). The reference genome of CBDRx (CsCBD), a high‐CBD variety with 15% CBD and 0.3% THC was sequenced and assembled recently (Grassa *et al*., 2018). The Medicinal Genomics group produced a 1.07Gb draft assembly representing Jamaican Lion DASH (CsJLD), a medical strain with 13% CBD and 9% THC (McKernan *et al.,* 2018). The genome assembly of another three marijuana varieties (Pineapple Banana Bubba Kush (CsPBB), LA Confidential (CsLAC), Chemdog91 (CsCD91)) with high THC (18–22%) and low CBD (<1%), and a medical marijuana strain, Cannatonic (CsCAN) with high CBD (15–22%) and high THC (6–10%) were sequenced and assembled by four cannabis research companies. In addition to the rapid accumulation of cannabis genome sequencing data, several transcriptomic, proteomic and metabolomic studies have uncovered the gene expression profiles, protein profiles and gene–metabolite relationships in individual varieties (Livingston *et al*., 2020; Vincent *et al*., 2019; Zager *et al*., 2019). However, an integrative functional genomic database of multiple varieties, enabling users to jointly examine and utilize relevant data, is lacking for cannabis.

We thus developed the first integrated functional genomics database for cannabis (CannabisGDB, https://gdb.supercann.net), including 5.84 Gb genome sequences of 8 cultivated cannabis plants downloaded from NCBI Assembly database. Additionally, a total of 195.7 Gb RNA sequencing (RNA‐seq) data cover 16 varieties downloaded from the NCBI SRA database and one sequenced by this study. Furthermore, 7 liquid chromatography mass spectral (LC‐MS) projects quantifying major cannabinoids were collected from literatures. We also collected 5 independent data sets of MS‐based proteomics in the literatures covering 4 tissues. After masking the repeat sequences, the genomic sequences were used to predict genes based on homology prediction of related species (*Mours alba*, *Humulus lupulus*, *Solanum lycopersicum* and *Arabidopsis thaliana*), transcriptional evidence together with ab initio gene prediction programs. In addition, we comprehensively annotated genes from different perspectives including Gene Ontology (GO), KEGG orthology (KO), non‐redundant (Nr) peptide database, UniProt protein database and PFAM domain database (Figure 1a).

**Figure 1:**
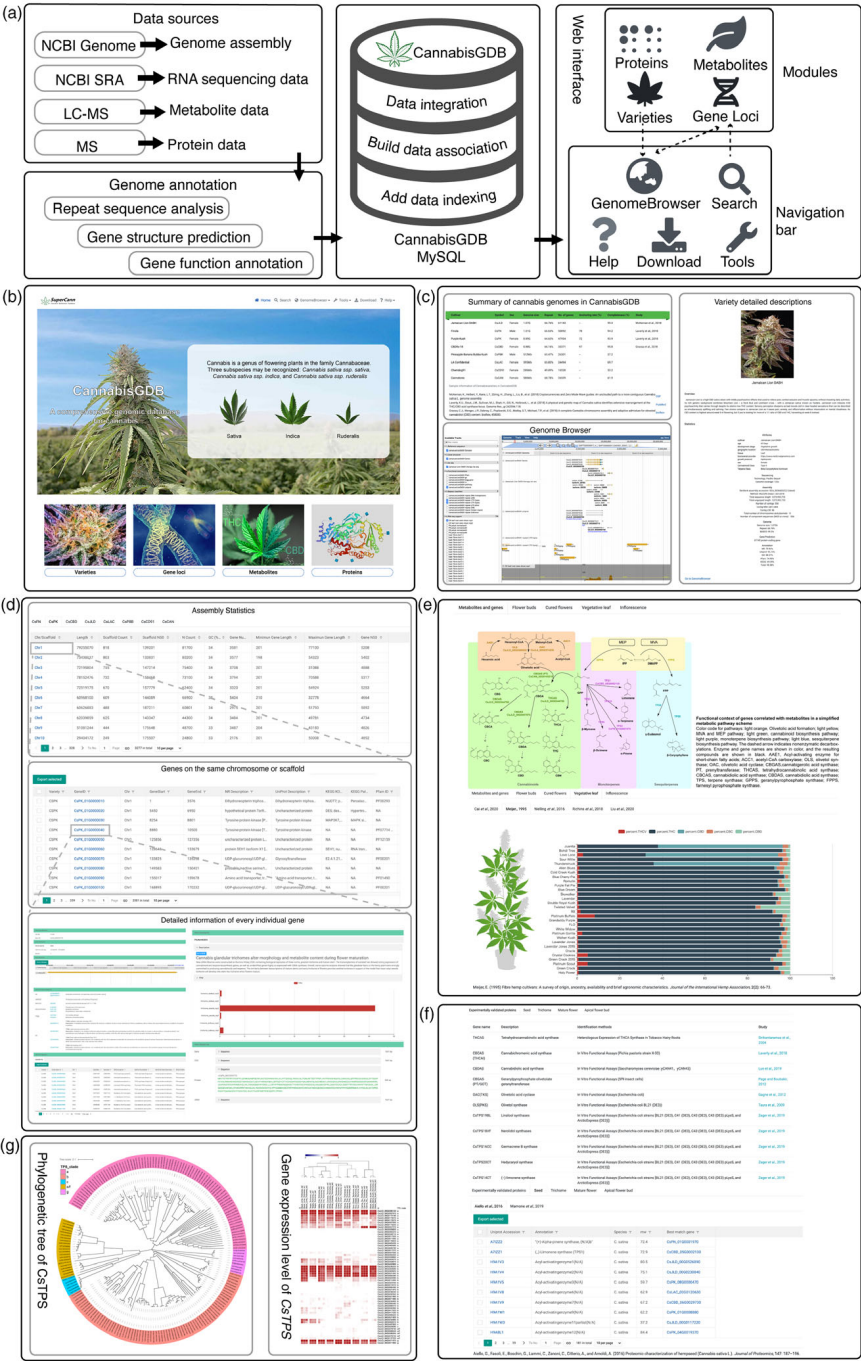
The schematic, screenshots of representative resources and a user case of CannabisGDB. (a) The flow diagram showing design and construction of CannabisGDB. (b)The home page of CannabisGDB. (c)The ‘varieties module’ providing summary of cannabis genomes, detailed information of cannabis varieties and genome browser tool. (d) The ‘gene loci module’ showing detailed information of genes identified in this study. (e) The ‘metabolites module’ providing chemical phenotypes in various cannabis varieties. (f) The ‘proteins module’ presenting information of experimentally identified proteins. (g) A case study for the application of CannabisGDB. Dashed lines indicate linkages between different pages.

CannabisGDB consists four modules: (1) varieties, (2) gene loci, (3) metabolites and (4) proteins (Figure 1b). The ‘varieties module’ presents 8 sequenced cannabis varieties with specific characteristics in terms of phenotype and genome assembly. Users can view a detailed description and image for each variety, together with the global statistics of the respective genome sequencing, assembly and the functional annotation for the protein‐coding genes. The genome browser attached to each genome displays a stack of aligned annotation tracks including functional annotation, gene structure, genome sequence, repeat sequence, Iso‐seq transcripts and gene expression (Figure 1c). The ‘gene loci module’ incorporates a total of 286 632 genes identified from the 8 cannabis varieties, together with chromosome or scaffold assembly statistics for each variety. The information regarding genomic position and multifaceted gene annotations for all genes on the same chromosome or scaffold can be viewed as organized sections by clicking the chromosome or scaffold. Furthermore, users can view the detailed information of every individual gene, such as gene ID, gene structure and gene orthogroups. Users can also obtain CDS, protein, cDNA and gene (including introns) sequence in FASTA format (Figure 1d). The ‘metabolites module’ hosts the chemical phenotype information of 210 varieties and provides multiple dynamic charts for users to visualize and investigate the major cannabinoids contents in four tissues including flower buds, cured flowers, vegetative leaf and whole inflorescence (Figure 1e). The ‘proteins module’ presents the users the list of proteins identified from seed, trichome, mature flower and apical flower bud (Figure 1f).

CannbiasGDB provides some popular bioinformatics tools for browsing, searching, analysing and downloading located in the navigation bar (Figure 1a, b). The ‘Search’ tool allows users to retrieve gene information by inputting specific format codes or keywords. The ‘Genome Browser’ tool provides a fast and interactive genome browser for navigating large‐scale high‐throughput sequencing data under a genomic framework. The ‘BLAST’ tool performs homology searches with different data sets of cannabis. ‘Primer3’ is the primer design tool. The ‘SynVisio’ tool is available for detection and evolutionary analysis of gene synteny and collinearity between cannabis varieties. The ‘Heatmap’ tool allows users to upload their genes of interest and create a heatmap. The ‘Enrichment’ tool performs GO or KEGG enrichment analysis on a given gene set showing maximum 20 enriched GO or KEGG terms. The ‘Download’ section allows users freely obtain all the data collected by CannabisGDB in batches. Furthermore, we provide a ‘Help’ section to familiarize the users with the database.

A typical user case picked from our web tests is present in Figure 1g: exploring cannabis *terpenes synthase* (*CsTPS*) gene family. There are 22 TPS proteins of cannabis in Uniprot annotated with two Pfam keywords, ‘PF1397 Terpene_synth’ and ‘PF03936 Terpene_synth_C’. Then, we searched CsTPSs in CannabisGDB with these two Pfam keywords. A total of 217 CsTPSs from 8 varieties was found, including 26 from CsCBD, 32 from CsFN, 58 from CsJLD, 48 from CsPK and 6 from CsLAC, 16 from CsPBB, 5 from CsCD91 and 26 from CsCAN. The phylogenetic tree using maximum likelihood method with 1000 bootstrap was constructed based on the protein sequences of the high‐quality genome assemblies (CsCBD, CsFN, CsPK and CsJLD) together with *Arabidopsis thaliana* TPSs defined as outgroups using MEGAX program. The *TPS* genes have been divided into three classes: Class I consists of *TPS‐c*, *TPS‐e/f* and *TPS‐h* (*Selaginella* specific); Class II consists of *TPS‐d* (*Gymnosperm* specific) and Class III consists of *TPS‐a*, *TPS‐b* and *TPS‐g* (Chen *et al*., 2011). Our results indicate that CsTPSs can be classified into two classes with 5 clades. Additionally, 6/7 of the CsTPSs are the members of Class III, and none of Class II was identified from these 4 Cannabis varieties. Among 5 clades, the TPS‐b clade is the largest (69 proteins), while the TPS‐c clade is the smallest (5 proteins). Interestingly, all the members in the TPS‐c clade were found on the sex chromosomes. Furthermore, we explored the expressions of *CsTPSs* among nine different cannabis varieties and presented as a heatmap (Figure 1g).

By integrating the genomic, transcriptomics, proteomics and metabolomics data, CannabisGDB can be used to assemble valuable information to facilitate basic, translational and applied research in cannabis. CannabisGDB is the first part of the SuperCann series database and will become a central gateway for the global cannabis community to better understand cannabis biology, thus benefitting the whole cannabis industry.

## Conflict of interests

The authors declare no conflict of interest.

## Author contributions

SC, YJZ, WT, SY and YL conceived and designed this research. SC, ZZ, SH, XB, ZH and LH performed the experiments. GH, SY and YL prepared the article.
